# A New Deep Dual Temporal Domain Adaptation Method for Online Detection of Bearings Early Fault

**DOI:** 10.3390/e23020162

**Published:** 2021-01-29

**Authors:** Wentao Mao, Bin Sun, Liyun Wang

**Affiliations:** 1 School of Information Engineering, Zhengzhou University of Industrial Technology, Zhengzhou 451100, China; 121114@htu.edu.cn (B.S.); zzwly0428@163.com (L.W.); 2 School of Computer and Information Engineering, Henan Normal University, Xinxiang 453007, China

**Keywords:** fault detection, deep learning, transfer learning, anomaly detection, bearing

## Abstract

With the quick development of sensor technology in recent years, online detection of early fault without system halt has received much attention in the field of bearing prognostics and health management. While lacking representative samples of the online data, one can try to adapt the previously-learned detection rule to the online detection task instead of training a new rule merely using online data. As one may come across a change of the data distribution between offline and online working conditions, it is challenging to utilize the data from different working conditions to improve detection accuracy and robustness. To solve this problem, a new online detection method of bearing early fault is proposed in this paper based on deep transfer learning. The proposed method contains an offline stage and an online stage. In the offline stage, a new state assessment method is proposed to determine the period of the normal state and the degradation state for whole-life degradation sequences. Moreover, a new deep dual temporal domain adaptation (DTDA) model is proposed. By adopting a dual adaptation strategy on the time convolutional network and domain adversarial neural network, the DTDA model can effectively extract domain-invariant temporal feature representation. In the online stage, each sequentially-arrived data batch is directly fed into the trained DTDA model to recognize whether an early fault occurs. Furthermore, a health indicator of target bearing is also built based on the DTDA features to intuitively evaluate the detection results. Experiments are conducted on the IEEE Prognostics and Health Management (PHM) Challenge 2012 bearing dataset. The results show that, compared with nine state-of-the-art fault detection and diagnosis methods, the proposed method can get an earlier detection location and lower false alarm rate.

## 1. Introduction

Early fault detection always plays a key role in the field of bearing prognostics and health management (PHM). In most recent years, the quick development of sensor techniques and artificial intelligence gave rise to a new problem: early fault online detection [1]. Compared with the traditional fault detection and diagnosis problems [2,3,4], early fault online detection is essentially a problem of anomaly detection with streaming data, that is the monitoring data of the target bearing arrive sequentially, and fault detection is conducted within a sampling interval. This new detection mode can evaluate the change of the working status of bearings in a very short time, avoiding economic losses caused by system halt. Obviously, early fault online detection should not only be sensitive to an early fault, but also be robust enough to avoid false alarms that are usually caused by running-in, lubrication, and so on. Especially, a false alarm can cause unplanned equipment shutdown, so online detection should pay more attention to avoiding false alarms rather than missing alarms. Such characteristics and requirements present a new challenge to the online detection method.

This paper mainly tackles early fault online detection in unsupervised mode, i.e., with no available state information for whole-life degradation data. For online scenarios, a straightforward solution is using the initial part of online data (regarded as the normal state) to construct a one-class classification model. However, a trustful model can usually be built waiting for a long enough time to get sufficient data for model training, especially for a deep neural network. One can certainly accumulate enough whole-life degradation data in an offline environment, e.g., a laboratory. However, the distribution drift between offline data and online data is inevitable due to the change of working conditions. In this scenario, the offline trained model cannot be directly applied to the online task. Therefore, how to transfer fault information (e.g., detection rules) between different working conditions has become a key issue to improve the accuracy and robustness of early fault online detection.

Presently, most traditional fault detection methods heavily rely on the fault features [5] extracted from vibration signals, such as wavelet features [6] and envelop spectrum features [7]. These features are then fed into a classification model such as support vector machine (SVM) [5], naive Bayesian [8], Fisher discrimination analysis [9], artificial neural network [10], and support vector data description (SVDD) [11]. In the past decade, deep learning techniques have been successfully introduced to bearing PHM due to their superior capability of end-to-end feature extraction. As the process of deep feature extraction is self-adaptive with no human intervention, various deep learning techniques have been successfully applied to the fault detection and diagnosis of different rotating machinery [12,13,14,15]. However, neither traditional machine learning methods, nor deep learning techniques can effectively solve the problem of distribution drift. Therefore, these methods are not applicable to online detection. According to the authors’ literature survey, very few works were found to conduct online anomaly detection. For instance, Lu et al. [16] utilized merely the initial part of online data to build a long short-term memory (LSTM) network and then recognized anomalies by calculating the residual error between real data and the LSTM prediction. Mao et al. [17] utilized semi-supervised SVM and a deep auto-encoder network to sequentially update the classification model for online detection. However, as this method merely used a small amount of normal state data to train the initial model, the extracted deep features are easily biased and then cause a false alarm.

From the discussion above, the most vital challenge to improve the performance of online detection is the effective transfer of fault knowledge between offline and online working conditions. As one of the research hotspots in machine learning, transfer learning aims to improve the predictive performance in one domain (called the target domain) by using the prior information contained in the data of another related, but different domain (called the source domain) [18]. As a kind of transfer learning technique, domain adaptation [19] focuses on the across-domain transfer of domain information. Domain adaptation can be realized well on deep neural networks by adaptively extracting domain-invariant feature representation [20]. Especially in the recent 2–3 years, deep domain adaptation has been applied to fault diagnosis [21,22,23] and remaining useful life prediction [24,25]. According to our literature survey, there are some preliminary research works [26] in the field of early fault detection. In these works, the role of deep domain adaptation is to learn fault information by leveraging the data from different working conditions. However, there still are some shortcomings: (1) most of these works need labeled data to train a classification model, which is not easy to realize in real-world applications; (2) most of these works mainly focus on anomalous samples rather than the temporal relationship between consecutive samples. As a result, the fault information cannot be sufficiently extracted, which may cause false alarms and reduce detection accuracy.

To solve such shortcomings, this paper proposes a new online detection method of bearing early fault based on deep transfer learning techniques. Specifically, this method contains an offline stage and an online stage. In the offline stage, a new state assessment method is firstly proposed by integrating the Hilbert–Huang transform (HHT) and support vector data description (SVDD) to determine the period of the normal state and the degradation state. The assessment results can provide the corrected data label for further domain adaptation. Furthermore, a new deep dual temporal domain adaptation (DTDA) model is proposed to extract temporal common fault information between different working conditions. In the online stage, the sequentially collected monitoring signals are directly fed into the DTDA model to recognize if a fault occurs. This process does not need a re-training model, since the domain-invariant feature representation has been extracted by the DTDA model. Finally, a set of comparative experiments is conducted on the IEEE PHM Challenge 2012 bearing dataset, and the results demonstrate the effectiveness of the proposed method.

The main contributions of this paper can be summarized as follows:

(1) This paper proposes a new dual temporal domain adaptation model with a dual adaptation strategy. Different from most current deep transfer learning techniques, this model can transfer temporal information of degradation sequences by integrating the time convolutional network (TCN) [27] and the domain adversarial neural network (DANN) [28]. This model can enlarge the temporal characteristics in domain-invariant feature representation and then raise the discrimination between the early fault feature and the normal state feature. According to the authors’ best knowledge, there are very few fault detection methods based on transfer learning with temporal information.
(2) This paper presents a new online health indicator (HI) construction method of bearings. This method adopts the temporal common features extracted by the DTDA model and uses principal component analysis (PCA) [29] to get a one-dimensional component. As the extracted common features are representative of the online working condition, the obtained HI can effectively describe the degradation process and provide an intuitive evaluation of online detection results.

This paper is organized as follows. In Section 2, a brief summary about TCN and DANN is provided. In Section 3, the details of the proposed method are elaborated. Section 4 is devoted to showing the experimental results on a widely-used bearing dataset, the IEEE PHM Challenge 2012 dataset, followed by the conclusion of this paper in the last section.

## 2. Background

### 2.1. Introduction of the TCN

**Figure 1:**
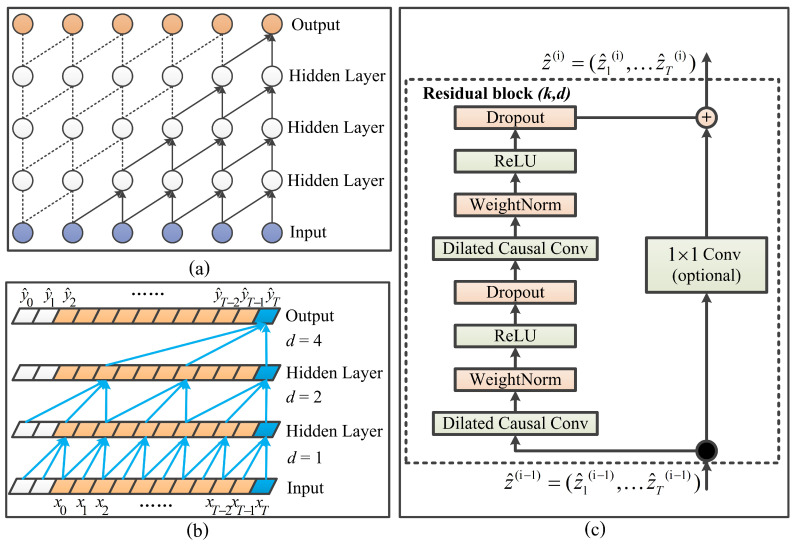
Structure of the time convolutional network (TCN) with (**a**) causal convolution, (**b**) dilated convolution, and (**c**) the residual module.

Rooted in the convolutional neural network (CNN) [30], the TCN has been proven equal to or even better than the recurrent neural network (RNN) [31] in dealing with temporal data [27]. The TCN is mainly composed of three parts: causal convolution, dilated convolution, and residual module, as depicted in Figure 1. A detailed introduction of each part will be given as follows.

Causal convolution is a one-way structure, i.e., the value at time *t* of the upper layer only depends on the value at time *t* and before time *t* of the next layer [27], as shown in Figure 1a. Causal convolution brings the time constraint structure. Dilated convolution is designed to solve the problem that the modeling length with temporal data is restrained by the size of the convolution kernel. In Figure 1b for example, the dilated factor *d* = 1 in the first layer means that every sample is calculated in convolution. If *d* = 4, all four samples are calculated together. As a result, the TCN can obtain a larger receptive field through fewer layers. Here, the function *F* of dilated convolution with the element *s* of the sequence *X* is shown as:

(1) $$F\left(s\right)=\left(X*df\right)\left(s\right)=\sum_{i=0}^{k-1}f(i)\cdot X_{s-d\cdot i}$$

where *f* is the convolution operation and *k* is the size of the convolutional kernel.

As shown in Figure 1c, the residual module contains two layers of dilated causal convolution and ReLU mapping. Besides, the TCN runs the dropout after each convolution layer to achieve regularization. The residual module can be expressed as:

(2) $${\hat{z}}^{\left(i\right)}={\hat{z}}^{\left(i-1\right)}+F\left(s\right)$$

### 2.2. Introduction of the DANN

**Figure 2:**
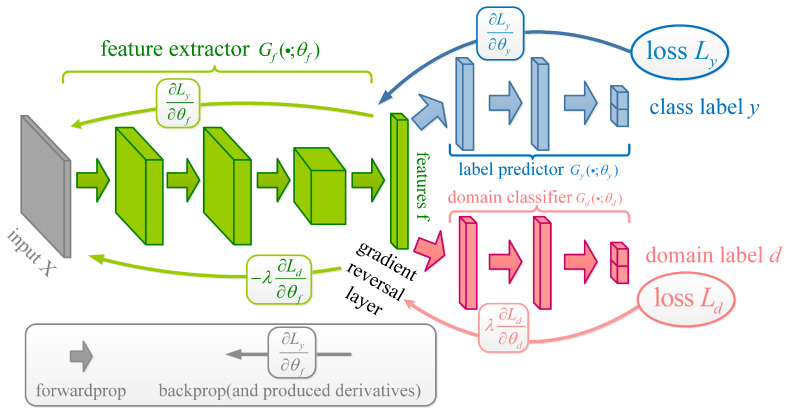
Schematic diagram of the domain adversarial neural network (DANN).

Proposed by Ganin et al. [28], the DANN has become an overwhelming domain adaptation model. The structure of the DANN is shown in Figure 2. In Figure 2, given source domain samples $\left(x_{i}^{s},y_{i}\right)$ and domain samples $\left(x_{i},d_{i}\right)$, the green part is a feature extractor $G_{f}\left(\cdot ;\theta_{f}\right)$, the blue part is a source domain classifier $G_{y}\left(\cdot ;\theta_{y}\right)$, and the red part is a domain classifier $G_{d}\left(\cdot ;\theta_{d}\right)$. The adversarial training strategy means that $G_{y}\left(\cdot ;\theta_{y}\right)$ can recognize the data from the source domain using the features extracted by $G_{f}\left(\cdot ;\theta_{f}\right)$ while ensuring that $G_{d}\left(\cdot ;\theta_{d}\right)$ cannot recognize from which domain the data come.

The training process of the DANN mainly concentrates on optimizing $G_{y}\left(\cdot ;\theta_{y}\right)$ and $G_{d}\left(\cdot ;\theta_{d}\right)$. Then, the loss function of $G_{y}\left(\cdot ;\theta_{y}\right)$ is:

(3) $$L_{y}^{i}\left(\theta_{f},\theta_{y}\right)=L_{y}\left(G_{y}\left(G_{f}\left(x_{i}^{s};\theta_{f}\right);\theta_{y}\right),y_{i}\right)$$

The loss function of $G_{d}\left(\cdot ;\theta_{d}\right)$ is:

(4) $$L_{d}^{i}\left(\theta_{f},\theta_{d}\right)=L_{d}\left(G_{d}\left(G_{f}\left(x_{i};\theta_{f}\right);\theta_{d}\right),d_{i}\right)$$

By adding a gradient reversal layer between $G_{y}\left(\cdot ;\theta_{y}\right)$ and $G_{d}\left(\cdot ;\theta_{d}\right)$, the total loss function of the DANN becomes:

(5) $$\begin{array}{cc} E(\theta_{f},\theta_{y},\theta_{d}) & =\sum_{i=1,2,\ldots N}L_{y}\left(G_{y}\left(G_{f}\left(x_{i};\theta_{f}\right);\theta_{y}\right),y_{i}\right)-\lambda \sum_{i=1,2,\ldots N}L_{d}\left(G_{d}\left(G_{f}\left(x_{i};\theta_{f}\right);\theta_{d}\right),d_{i}\right)\\ \; & =\sum_{i=1,2,\ldots N}L_{y}^{i}\left(\theta_{f},\theta_{y}\right)-\lambda \sum_{i=1,2,\ldots N}L_{d}^{i}\left(\theta_{f},\theta_{d}\right) \end{array}$$

Using a back-propagation optimization algorithm like the stochastic gradient descent (SGD) algorithm, Equation (5) can be minimized to reach equilibrium. Then, a domain-invariant feature representation can be determined. The test data can then be classified using $G_{y}\left(G_{f}\left(x_{i};\theta_{f}\right);\theta_{y}\right)$.

## 3. Proposed Approach of Early Fault Online Detection

It is worth noting that the proposed method is unsupervised, i.e., all training data are whole-life degradation sequences with no state labels. The training data include a sufficient amount of data from offline working conditions (for example, in a laboratory) and a small amount of data from online working conditions (for example, in a real application). The goal of the proposed method is to recognize the occurrence of an early fault in the online data of a target bearing.

**Figure 3:**
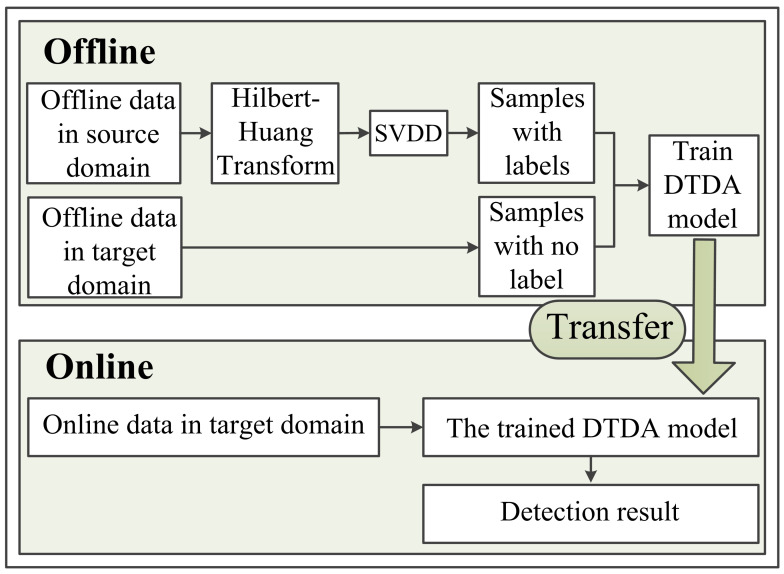
Flowchart of the proposed online detection method of early fault. SVDD, support vector data description; DTDA, dual temporal domain adaptation.

To reach this goal, the proposed method contains an offline stage and an online stage. In the offline stage, it needs to first grab the label information of offline data via state assessment and then extract the domain-invariant feature representation between the offline and online working conditions. Following this idea, a new state assessment method and a novel deep domain adaptation model named DTDA are proposed. In the online stage, the sequentially collected data batch is simply fed into such a feature representation to get the discriminative features for the online tasks and directly get the detection results. The steps stated above are shown in Figure 3.

### 3.1. State Assessment

The offline data are a whole-life degradation sequence, so we need to determine the label information of the normal state and early fault before conducting domain adaptation. Therefore, an efficient state assessment method is first presented in this section. Given a raw vibration signal sequence of a rolling bearing, the steps of the state assessment are as follows:

(1) Obtain the HHT [32] marginal spectrum data from the raw signal. First, decompose the raw signal x(t) as: $x\left(t\right)=\sum_{i=1}^{k}c_{i}\left(t\right)+r_{k}\left(t\right)$, where $c_{i}\left(t\right)$ is the *i*-th intrinsic mode function (IMF) component and $r_{k}\left(t\right)$ is the residual term. Second, run the Hilbert transform for each IMF component: $H\left[x\left(t\right)\right]=\frac{1}{\pi }\int_{-\infty }^{+\infty }\frac{x(\tau )}{t-\tau }d\tau$, and get the corresponding analytic signal: $C_{i}^{A}\left(t\right)=c_{i}\left(t\right)+jc_{i}^{H}\left(t\right)=a_{i}\left(t\right)e^{j\theta_{i}\left(t\right)}$, where $c_{i}^{H}\left(t\right)=\frac{1}{\pi }\int_{-\infty }^{+\infty }\frac{c_{i}\left(s\right)}{t-s}ds$, $a_{i}\left(t\right)=\sqrt{{c_{i}}^{2}+{\left(c_{i}^{H}\right)}^{2}}$, $\theta_{i}\left(t\right)=arctan\left(c_{j}^{H}/c_{i}\right)$, with the instantaneous frequency $\omega =\frac{d\theta (t)}{dt}$. Third, calculate the Hilbert spectrum: $H\left(\omega ,t\right)=\sum_{i=1}^{n}a_{i}\left(t\right)e^{j\theta_{i}\left(t\right)}$, and obtain the marginal spectrum: H(ω)=∫H(ω,t)dt.
  Here, the HHT is regarded as a signal processing method, as the HHT has two merits for analyzing signals: (1) no need to preset the orthogonal basis and (2) the good capability of processing the non-stationary signal. Therefore, here, the HHT is chosen as the signal processing method.
(2) Select the initial 500 samples of the whole-life degradation sequence. Set the HHT marginal spectrum of these samples as the normal state data, and train an SVDD model. Specifically, the optimization target of the SVDD is:
  (6) $$\begin{array}{c} \min_{a,R,\xi }R^{2}+C\sum_{i=1}^{n}\xi_{i}\\ s.t.{∥\phi (x_{i}-a)∥}^{2}\leq R^{2}+\xi_{i},\xi_{i}\geq 0,\forall i=1,2,\ldots n \end{array}$$
  where ξ is the slack variable, *R* and *a* are the radius and center of the hyper-sphere, and *C* is the regularization parameter.
(3) Select the sample sequentially from the beginning and feed the spectrum of the sample into the obtained SVDD. Calculate the distance between this sample and the hyper-sphere center of the SVDD:
  (7) $$d=\sqrt{K\left(x_{test},x_{test}\right)-2\sum_{i=1}^{n}\alpha_{i}K\left(x_{test},x_{i}\right)+\sum_{i=1}^{n}\sum_{j=1}^{n}\alpha_{i}\alpha_{i}K\left(x_{i},x_{j}\right)}$$
  where $K(x_{i},x_{j})$ is the kernel function and $\alpha_{i}$ is the Lagrange coefficient. If d≤R, the sample $x_{test}$ is recognized as in the normal state, otherwise it is a fault sample. As a result, the boundary between the normal state and the fault state can be determined.

### 3.2. Proposed DTDA Model

To realize an effective domain adaptation between offline and online working conditions, the DANN is chosen as the baseline algorithm. The DANN adopts the adversarial training strategy and can get a better domain-invariant feature representation even between quite different domains [28]. As training the DANN requires label the information of the source domain, the results of the state assessment presented in Section 3.1 can be used. Different from mature fault data, early fault data generally have a temporal characteristic that reflects the degradation process from the normal state to the fault state. More importantly, the degradation part of the early fault is similar between different bearing sequences [1]. Therefore, the effect of domain adaptation by the DANN can be further enhanced by extracting common temporal information. To extract temporal information well, the TCN is adopted as the feature extractor in the classical DANN, and we propose a new DTDA model.

Specifically, a strategy of dual adaptation is proposed in the DTDA model. This strategy comes from the following observations: (1) Different domains have different requirements on the amount of temporal information, e.g., degradation length. Then, the TCN may perform poorly due it not having a sufficiently large receptive field. (2) The adversarial training strategy used in the DANN may perform unstably when tackling the data with a large distribution difference. Following this analysis, an adaptation layer with the maximum mean discrepancy (MMD) [33] is first added after the TCN’s residual blocks. This layer can shrink the distribution difference of temporal features between the source domain and the target domain to some extent. Then, the DANN is run based on such adapted TCN features and can improve the stability of the DANN training as well.

**Figure 4:**
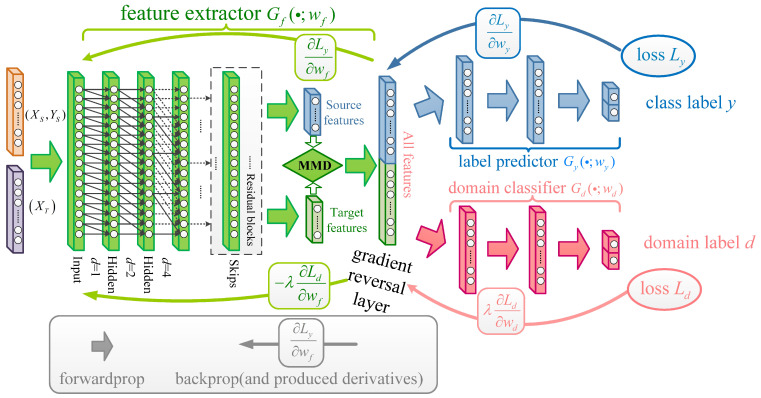
Structure diagram of the proposed DTDA model.

The above idea is shown in Figure 4. Specifically, the orange part represents the source data with labels that are obtained by the state assessment in Section 3.1. The purple part represents the available input data in the target domain. The green part is the feature extractor using the TCN, linked by an MMD adaptation layer. The blue part is the source domain label classifier that aims to recognize the normal state data from the fault data. The pink part is the domain classifier whose task is to discriminate the source domain data and the target domain data. The blue part and pink part are the same as the ones in Figure 2.

The training process of the DTDA model can be summarized as follows:

Step 1. Initialize randomly the weight *w* and bias *b*.

Step 2. Combine the source domain data and the target domain data as a whole, and feed them into the TCN to get the output:

(8) $$H_{1}=G_{f}\left(\sum_{i=1}^{m+n}w_{i}x_{i}-b\right)$$

where *m* and *n* are the sample number in the source domain and target domain, respectively, and $G_{f}\left(\cdot \right)$ is the feature extractor of the TCN.

Step 3. Denote by $X^{s}$ and $X^{t}$ the source domain feature and target domain feature in $H_{1}$, respectively. Then, realize the domain adaptation for $X^{s}$ and $X^{t}$ by using an MMD layer. The definition of the MMD is as follows:

(9) $$MMD\left(X^{s},X^{t}\right)={∥\frac{1}{m}\sum_{i=1}^{m}\phi (x_{i}^{s})-\frac{1}{n}\sum_{j=1}^{n}\phi (x_{j}^{t})∥}_{H}$$

where the function $\phi \left(\cdot \right)$ indicates a nonlinear mapping to a reproducing kernel Hilbert space (RKHS) and the subscript H refers to this RKHS.

Step 4. Denote by $X_{MMD}^{s}$ the source domain feature set adapted by the MMD layer. Feed $X_{MMD}^{s}$ into the source domain label classifier $G_{y}\left(\cdot \right)$ in Figure 4, and get the output $G_{y}\left(X_{MMD}^{s};w_{y}\right)$. The loss function of $G_{y}\left(\cdot \right)$ can be expressed as:

(10) $$L_{y}^{i}\left(w_{f},w_{y}\right)=L_{y}\left(G_{y}\left(X_{MMD}^{s};w_{y}\right),y_{i}\right)$$

where $w_{f}$ is the model parameter of the feature extractor composed of the TCN and MMD adaptation layer and $w_{y}$ is the model parameter of the source domain label classifier.

Step 5. Feed the adapted feature set $X_{MMD}$ of the source and target domains into the domain classifier $G_{d}\left(\cdot \right)$, then get the output: $G_{d}\left(X_{MMD};w_{d}\right)$. The loss function of $G_{d}\left(\cdot \right)$ can be expressed as:

(11) $$L_{d}^{i}\left(w_{f},w_{d}\right)=L_{d}\left(G_{d}\left(X_{MMD};w_{d}\right),d_{i}\right)$$

where $w_{d}$ is the model parameter of the domain classifier.

Step 6. After combining Equations (9)–(11), the optimization function of the DTDA model is:

(12) $$E\left(w_{f},w_{y},w_{d}\right)=L_{y}^{i}\left(w_{f},w_{y}\right)-\lambda L_{d}^{i}\left(w_{f},w_{d}\right)+\mu MMD\left(X^{s},X^{t}\right)$$

where λ,μ>0 are the regularization parameters, which are used to tune a trade-off between these three quantities during the learning process. Specifically, the larger the value of μ is, the higher the requirement for extracting common features is, and vice versa. Similarly, if the value of λ becomes smaller, the effect of the domain classifier is equivalent to being enhanced, and correspondingly, the samples are more difficult to recognize from the source domain or the target domain. In Section 4, a reverse cross-validation approach, which was adopted in the original DANN model [28], is employed to update the regularization parameters λ and μ. It is worth noting that the minus sign in Equation (12) means gradient reversal for reducing the distribution difference between source domain features and target domain features.

Another thing that needs to be noticed is that the classifier parameters of $w_{y}$ and $w_{d}$ are optimized in order to minimize their error on the training set; the feature extractor parameter of $w_{f}$ is optimized in order to minimize the loss of the source domain label classifier and to maximize the loss of the domain classifier; and the SGD algorithm is employed to minimize Equation (12) to update all three of them.

Step 7. In the training process, if the iteration number reaches a pre-defined number ρ or the difference between two consecutive training errors is less than a pre-defined threshold, the training is terminated; otherwise, go to Step 6.

After dual adaptation, i.e., MMD adaptation and the DANN, the feature distribution of the source domain data and target domain data tends to be consistent. After reaching convergence, the DTDA model can extract the common temporal feature representation of different domain data. In the experiment of this paper, the offline working condition is set as the source domain, and the online working condition is set as the target domain, then such a common feature representation can provide a channel to transfer fault information from the offline data to the online task.

### 3.3. Online Detection

Once the DTDA is trained, the common temporal feature representation can be obtained. The source domain label classifier $G_{y}\left(\cdot \right)$ can then be used well to recognize the fault in the data of the target domain. Therefore, in the online stage, the sequentially collected data batch is directly fed into $G_{y}\left(\cdot \right)$ to determine if a fault has occurred. This process does no need to re-train the DTDA, while the main computational cost is the linear calculation in $G_{y}\left(\cdot \right)$. As a result, the detection speed is very fast.

### 3.4. HI Construction

To intuitively evaluate the reliability of the detection results, a new HI construction method is also proposed based on the DTDA model. This method is simple and effective. Specifically, through feeding sequentially the online data batch into $G_{y}\left(\cdot \right)$, not only the detection results, but also the temporal features of the target bearing can be obtained. After detecting all the online data, the features of the whole degradation sequence can be obtained. Then, PCA is run to get the first principal component. After the smoothing operation, the obtained feature sequence is the HI of the target bearing. Since the feature extractor in the DTDA model can extract domain-invariant feature representation with strong discriminative ability, the HI constructed based on such a feature representation can be more sensitive to reflect various state changes of the target bearing. Certainly, the obtained HI can also verify the reliability of the online detection results.

## 4. Experimental Results

To verify the effectiveness of the proposed method, a set of comparative experiments is run on the IEEE PHM Challenge 2012 bearing dataset [34] in this section. The programming environment was Python 3.6 and MATLAB R2014. The experiments used the Windows operating system (OS) with an i5-7300 processor and 8 G memory.

### 4.1. Dataset Description

**Figure 5:**
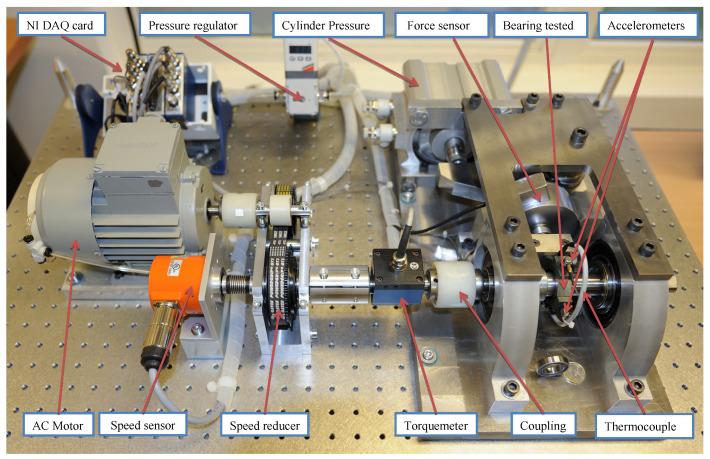
PRONOSTIAtest platform [34].

The IEEE PHM Challenge 2012 dataset was collected from PRONOSTIAtest platform, as shown in Figure 5, on which an accelerated degradation experiment was conducted to collect run-to-failure data within a few hours. The PRONOSTIA platform is composed of three parts: rotating part, load part, and data collection part. The rotating part has a motor with a power of 250 W. To accelerate degradation, the load part provides a 4000 N load for the rolling bearing. Vibration signals were collected using an accelerometer sensor placed in the horizontal direction. The sampling frequency was 25.6 kHz, while the data were recorded every 10 s. In total, seventeen bearings were selected to collect whole-life degradation data under three working conditions. The specific information of the working conditions is shown in Table 1.

In this experiment, the seven bearings (i.e., Bearing1_1 to Bearing1_7) under the first working condition were selected as the source domain data. Moreover, Bearing 2 and Bearing 3 under the second working condition (i.e., Bearing2_2 and Bearing2_3) were taken as the offline data in the target domain, and we took Bearing 1 (i.e., Bearing2_1) and Bearing 4 (i.e., Bearing2_4) under the second working condition as the target bearings to be tested in the target domain.

### 4.2. Results of State Assessment

In this section, the results of the state assessment are provided. Taking Bearing 1_1 as an example, HHT was first run to get the marginal spectrum data for this bearing, and then, we chose the first 500 samples to train an SVDD model. The Gaussian radial basis function (RBF) kernel was adopted, and the regularization parameter and kernel parameter of SVDD were set to one and 0.001, respectively. After feeding the HHT spectrum data into the trained SVDD model sequentially, the results of the state assessment can be obtained. Table 2 shows the period of the normal state and the fault state of all seven bearings under the first working conditions. These results will be used as the label information for training a DTDA model in the next section.

### 4.3. Results of Online Detection

In this section, Bearing2_1 and Bearing2_4 under the second working condition are chosen as the target bearings to evaluate the effectiveness of the proposed method. Specifically, these two bearings have quite different degradation trends and noise levels in the normal state. Bearing2_1 has a long period of slow degradation, while Bearing2_4 has no apparent early fault state and quickly evolves to the fast degradation state. Therefore, these two bearings are believed to be representative enough to provide a comprehensive evaluation.

#### 4.3.1. Results of Bearing2_1

**Figure 6:**
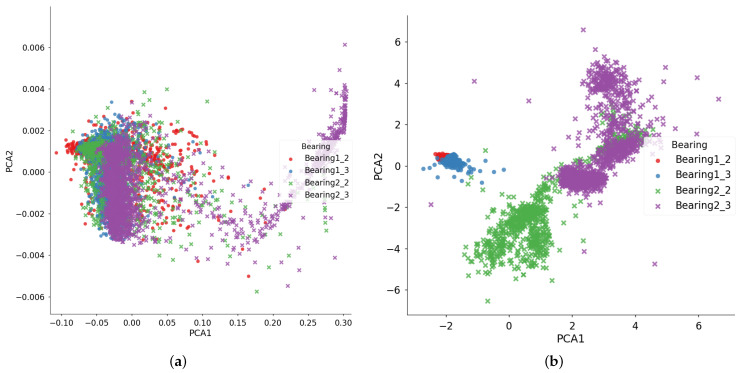
Feature distribution of the four bearings under the first and second working conditions extracted by (**a**) the DTDA and (**b**) the deep autoencoder (DAE). Here, PCA is used for visualization.

First, Figure 6 provides the visualized feature distribution after domain adaptation by DTDA. Here, two bearings (Bearing1_2 and Bearing1_3) in the source domain and two bearings (Bearing2_2 and Bearing2_3) in the target domain are chosen. For comparison, Figure 6 also provides the feature distribution by using the deep autoencoder (DAE) without domain adaptation. Here, PCA is used for visualization. From Figure 6b, before domain adaptation, the feature distribution of the bearings in the source domain (red points and blue points) and the target domain (purple points and green points) vary largely, which indicates that different working conditions have different data distribution characteristics. However, after domain adaptation by the DTDA, the feature distribution of different domains tends to be consistent, as shown in Figure 6a. The results shown in Figure 6 demonstrate that the DTDA model can effectively extract domain-invariant feature representation between different working conditions.

**Figure 7:**
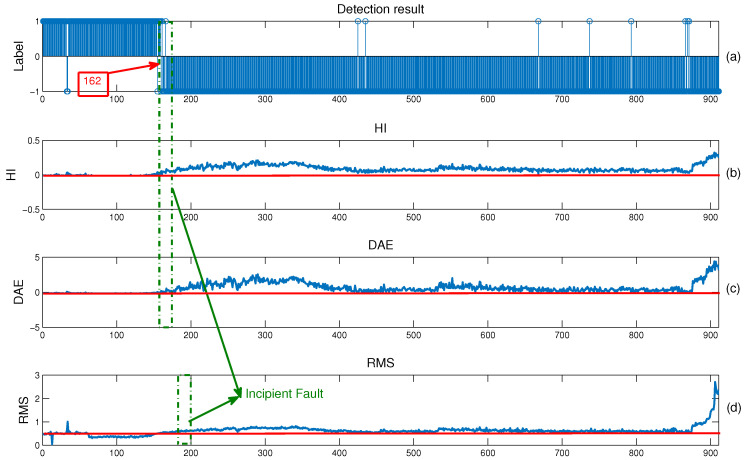
Online anomaly detection results on Bearing2_1 using (**a**) the proposed method, (**b**) the constructed health indicator (HI) by PCA, (**c**) the constructed HI by DAE, and (**d**) the RMS curve. Here, the label “1” in Subfigure (**a**) indicates the normal state; “−1” indicates the fault state.

Second, Figure 7 provides the results of early fault online detection on Bearing2_1. To ensure the results are more reliable, the location of five successive anomalous samples is defined as the occurrence of an early fault. The anomaly before the occurrence location is defined as a false alarm. For straightforward comparison, Figure 7 also reports the HI sequence built by the proposed method in Section 3.4 and the root mean square (RMS) curve. From Figure 7a, an early fault occurs at Sample 162 with only four false alarms. From Figure 7b, the HI sequence has a basically consistent trend with Figure 7a, which proves that the HI sequence can be used to evaluate the reliability of the detection results. As a widely-used indicator to reflect the degradation trend, the RMS curve rises slowly at Sample 180, which lags by nearly 20 samples. This comparison demonstrates that the domain-invariant feature representation extracted by the DTDA model has a better discriminative ability.

The effectiveness of the obtained HI sequence is further analyzed. In Section 3.4, PCA is used to shrink the degradation features into a one-dimensional component, which performs as an HI. To test the effectiveness of PCA in HI construction, the DAE is also introduced to build an HI sequence by replacing PCA, as shown in Figure 7c. Specifically, after extracting the domain-invariant feature representation, the features of Bearing2_2 and Bearing2_3 in the target domain can be generated. Then, a DAE model with one-dimensional output is trained using the feature set of these two bearings. Finally, the online features of Bearing2_1 are directly fed into the obtained DAE model to get a one-dimensional output, i.e., the expected HI sequence. It is clear that the two HI sequences built by PCA and DAE are nearly identical in geometric shape, and the location of the early fault is almost the same. This phenomenon indicates that the common features obtained by the DTDA have good representative capability to reflect the degradation trend, while both PCA and the DAE can easily extract a representative component from the features to build the HI. Still, the HI by PCA is a little more sensitive to the early fault. Since training a DAE model generally needs a sufficient amount of data, less samples in the online stage may cause over-fitting. Moreover, the DAE is trained by a gradient descent algorithm, which has more computational cost than PCA. Under comprehensive consideration, PCA is believed to be more suitable for HI construction than the DAE.

In Figure 7d, the RMS curve fluctuates drastically in the initial part, even locating in the normal state. This phenomenon is mainly caused by the irregular vibration of running-in, assembly errors, etc., not by early fault. If the features are not representative (like RMS), there will be many false alarms in the normal state. Quite different from the RMS curve, the HI sequence has almost no irregular fluctuations in the normal state. This phenomenon shows that the DTDA model can extract fault features that are robust to the irregular fluctuations in the normal state. Moreover, the HI sequence has an obvious upward trend after the location of the early fault, while the RMS curve keeps flat for a long period. It is clear that the features extracted by the DTDA are more sensitive to early fault than the RMS feature. As a result, the DTDA model can generate deep features with better discriminative ability, which is helpful to improve the performance of early fault detection.

**Figure 8:**
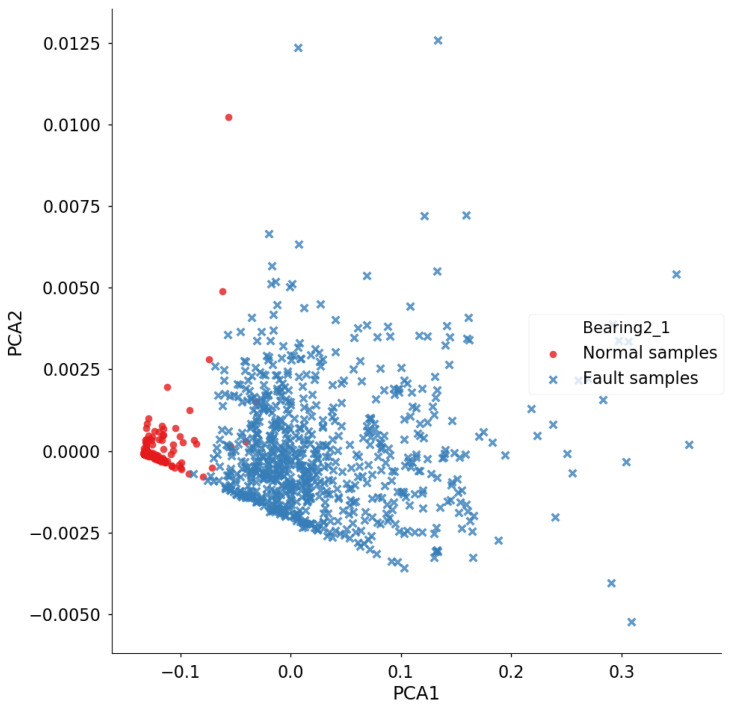
Online features of the target bearing Bearing2_1 in the IEEE PHM Challenge 2012 dataset.

To further analyze the comparative advantage of the proposed method, the trained DTDA in the offline stage is used to generate the online features of Bearing2_1, as shown in Figure 8. It is worth noting that the visualization label in Figure 8 corresponds to the results in Figure 7a. The features of the two states are almost linearly separable, which indicates that the features extracted by DTDA are discriminative for early fault and very applicable for online detection.

#### 4.3.2. Results of Bearing2_4

**Figure 9:**
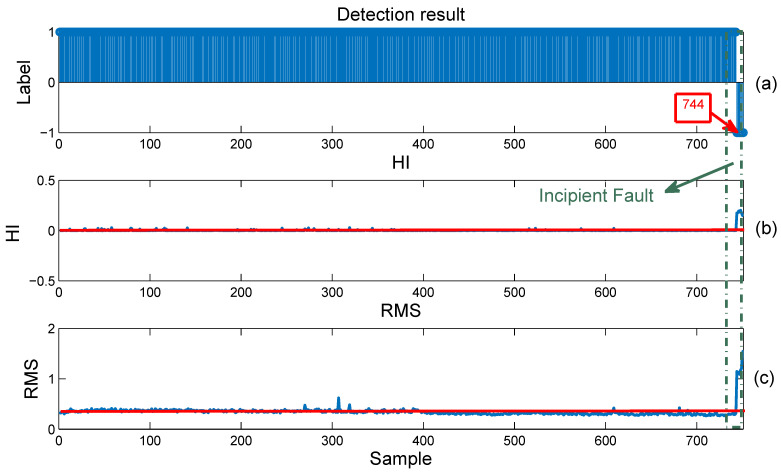
Online anomaly detection results for Bearing2_4 using (**a**) the proposed method, (**b**) the constructed HI, and (**c**) the RMS curve. Here, the label “1” in Subfigure (**a**) indicates the normal state; “−1” indicates the fault state.

First, similar to Figure 7, Figure 9 shows the results of the online detection for Bearing2_4. This bearing falls into the early fault state at Sample 744, with no false alarm. It is also clear that the trend of the HI sequence and the RMS curve completely matches the detection results in Figure 9a, as shown in the dotted frame. Moreover, compared with the RMS curve, the HI sequence does not have an obvious fluctuation, which proves the effectiveness of the proposed method in early fault online detection.

**Figure 10:**
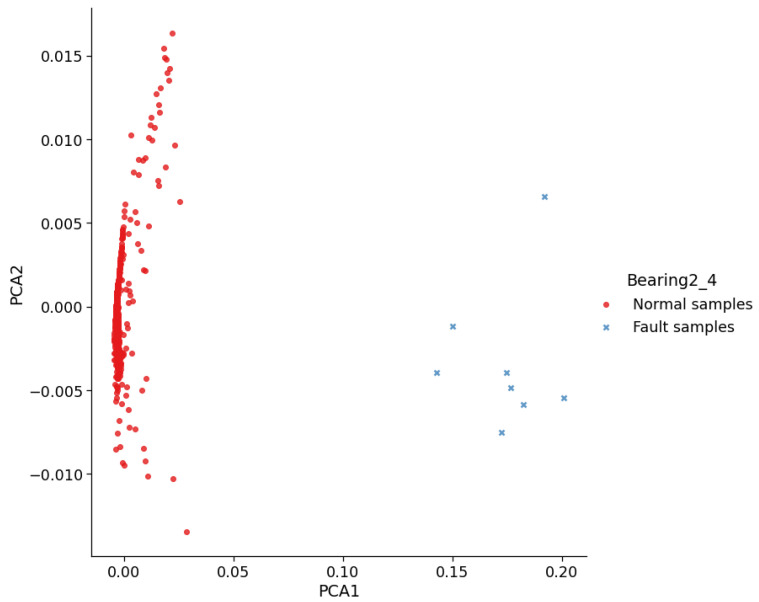
Online features of the target Bearing2_4. Here, PCA is used for visualization.

Figure 10 further shows the online features of Bearing2_4, which were generated using the domain-invariant feature representation extracted by the DTDA. The separability of these features is even more obvious than the online features of Bearing2_1 shown in Figure 8. This phenomenon is caused by the degradation process of Bearing2_4 in which the bearing transitions directly from the normal state to the fault state. In this scenario, the fault state data are certainly easy to distinguish from the normal state data. Figure 10 proves again that the proposed method can effectively recognize the normal state and the early fault state.

### 4.4. Comparative Results with State-of-the-Art Methods

In this section, nine state-of-the-art methods of bearing fault detection are introduced for a comprehensive comparison. These nine methods include one typical signal analysis method (Method 1), five anomaly detection methods without transfer learning (Methods 2–6), and three anomaly detection methods with transfer learning (Methods 7–9). For simplicity, the proposed method is named DTDA.

Following [16], two evaluation metrics are employed: (1) the detection location, which is the location (number) of the signal snapshot of the appearing fault; (2) the number of false alarms, which is the number of anomalies before the detection location. The comparative results are reported in Table 3.

From Table 3, the proposed method DTDA obtains the earliest detection location and almost the lowest number of false alarms. Although RD-DTL and SDFMhave a lower number of false alarms than the DTDA, the detection location of these two methods is relatively late. It is worth noting that the detection locations of all ten methods on Bearing2_4 are not much different. This is because the bearing evolves quickly from the normal state to the fast degradation state, with a very short period of early fault. Since the data of the fast degradation state are quite different from the normal state data, all methods can detect faults at the location of the state change. However, the number of false alarms produced by different methods on Bearing2_4 is not the same. Some methods like iFOREST and the local outlier factor (LOF) produce too many false alarms. Moreover, bandwidth empirical mode decomposition-adaptive multi-scale morphological analysis (BEMD-AMMA) has no false alarm since it utilizes signal analysis to conduct fault detection by observing the fault frequency.

Here, a detailed analysis of the comparative results is listed as follows:

(1) Comparison with BEMD-AMMA:
  BEMD-AMMA can be viewed as a state-of-the-art signal analysis-based method for bearing early fault detection. This method first uses the bandwidth empirical mode decomposition (BEMD) to reconstruct the raw vibration signal and then utilizes an adaptive multi-scale morphological analysis (AMMA) algorithm to demodulate the reconstructed signal to obtain time-domain signals. Finally, a fault can be determined if the fault characteristic frequency can be observed. To calculate the fault characteristic frequency, this method has to know the various parameters of the target bearing and working condition in advance. Obviously, this limitation is too strict to achieve in the online scenario. Moreover, a fault only evolves to a certain degree, and the corresponding characteristic frequency can then appear. Therefore, the detection location will be delayed. In contrast, benefiting from the sensitivity of the early fault features extracted by the DTDA model, the proposed method can detect fault occurrence at an earlier location.
(2) Comparison with LOF:
  The LOF is a typical anomaly detection algorithm running on sample density. In this experiment, the first 100 samples at the starting online stage were chosen to calculate the LOF value, and then, the largest value is selected as the alarm threshold. The parameter *K* in the LOF was set to 10. From Table 3, the detection location of the LOF is later than that of the proposed method, while the number of false alarms is much larger. This is because the normal state of a bearing may have unexpected irregular fluctuations. Moreover, when using normal state data to train the LOF, the threshold value will be relatively larger, resulting in a late detection location.
(3) Comparison with iFOREST:
  iFOREST is also a typical anomaly detection algorithm adopting a random segmentation strategy. iFOREST segments all samples into various isolated outliers, and the ones with a shorter path are viewed as anomalies. In this experiment, the number of trees was set to 100. From Table 3, the detection location of iFOREST is much delayed for Bearing2_1, and too many false alarms appear. This is because with online samples arriving sequentially, the segmentation number continues to increase. Consequently, the detection performance is not stable.
(4) Comparison with SRD:
  SRD is a state-of-the-art early fault detection algorithm based on probability density estimation. In the offline stage, this method directly uses the original signals of multiple working conditions to establish a global model and determine the alarm limit. The online signals are fed into this model to get the detection results. However, this method does not take into account the difference between the bearing data under different working conditions. The setting of the alarm limit is also too subjective. Therefore, the detection location was very much delayed for Bearing2_1. In contrast, the proposed method does not need to manually set a threshold for detection, and the DTDA model can effectively transfer fault information between different working conditions to improve the detection performance.
(5) Comparison with SDFM:
  SDFM is a state-of-the-art online detection method for bearing early fault. This method employs a sliding window to determine the location of early fault occurrence by means of DAE features. The DAE network structure was set to [800, 512, 10], and the size of the sliding window was set to 100. Benefiting from the sliding window, this method had no false alarm, but the detection location was a bit delayed. The main reason is that this method is not a transfer learning method and heavily relies on the amount of offline training data. If the amount of training data is insufficient, the early fault information could not be extracted completely. In contrast, the proposed method can borrow data from offline working conditions to supplement early fault information for online detection.
(6) Comparison with S4VM-SODRMB:
  This method is also a state-of-the-art online detection method for bearing early fault. This method only needs online data to update model training in an unsupervised learning architecture. In this experiment, the first 100 samples of online data were accumulated to extract the DAE features and then train an initial SVM model. The sequentially collected data batch was used to update the SVM model successively. The radius-margin upper bound of leave-one-out error was then utilized to calculate an index for online detection. In Table 3, the detection location of this method was much delayed for Bearing2_1. The reason is that only a small amount of online data was used to train an initial model. Once the data contain irregular fluctuation or colored noise, the initial model will be biased, and the detection results will deteriorate. In contrast, the proposed method can utilize offline data to facilitate online detection. The transfer learning technique in the DTDA model guarantees the effective use of early fault information from offline data.
(7) Comparison with TCA + SVDD:
  Transfer component analysis (TCA) is a widely used transfer learning algorithm by minimizing the MMD distance between different domains. In this experiment, TCA was first run to conduct domain adaptation between the available data from offline and online working conditions. Then, the common features were used to train an SVDD model by using the available training data from the online working condition. This SVDD model was used to recognize anomalies in the online stage. In this experiment, the regularization parameter and kernel parameter were set to 10 and one, respectively. From Table 3, it is clear that the detection location of TCA+SVDD was much delayed for Bearing2_1, and the number of false alarms became larger. The reason is that TCA conducts domain adaptation with a shallow model, while such domain adaptation is in a single mode. In contrast, the proposed method not only conducts dual domain adaptation, but also extracts common temporal information of early fault. Therefore, the proposed method can provide more representative features for online detection.
(8) Comparison with RD-DTL and OD-DTL:
  RD-DTL and OD-DTL are both the newest state-of-the-art early fault online detection methods based on deep transfer learning. RD-DTL first uses a robust auto-encoder to determine the periods of different degradation states and then constructs an MMD-based DAE network to extract common features for the normal state, followed by an SVM model for recognition. This method focuses on the robustness of the online detection model. OD-DTL utilizes a pre-trained VGG-16 network on the ImageNet dataset to fine-tune a deep neural network for bearing online detection. This method only conducts model-level domain adaptation, not considering the feature transfer. Therefore, the performance of fault detection is limited. From Table 3, these two methods get similar results as the proposed method. RD-DTL even gets zero false alarms. However, the proposed method gets an earlier detection location than them. The main reason is that the proposed method conducts dual domain adaptation with temporal information. Therefore, the online features by DTDA are more sensitive to early fault.

In summary, the proposed DTDA model can achieve dual domain adaptation at the feature level, which can facilitate the transfer of fault information between different working conditions. Moreover, the DTDA utilizes the TCN as the feature extractor to extract the temporal information of the degradation process, which can improve the representative ability of the online features for early fault. Therefore, the proposed method is more applicable to the online detection of bearing early fault.

Another problem of online detection is computational time. The proposed method needs to train the DTDA model in the offline stage and then directly inputs the sequentially collected online data into the model to recognize the fault occurrence. The dual domain adaptation by the DTDA provides a domain-invariant feature representation with a better discriminative ability for the online task. The offline model training is computationally expensive, since the adversarial training of the DTDA is an iteration process. However, no additional training time is required in the online stage. The classification on an online sample by the trained DTDA model is almost a linear operation, so the time for recognizing an online sample is very short. For this reason, the corresponding time data are not provided in Table 3.

## 5. Conclusions

Online detection of bearing early fault is an application-oriented fault detection method with significant practical meaning. This paper proposes a new online detection method of early fault based on deep dual temporal domain adaptation. This method adopts deep domain adaptation with temporal information to extract domain-invariant feature representation with stronger discriminative ability. Employing this representation as the channel of information transfer, the proposed method can improve the detection robustness and accuracy in the online scenario with fewer false alarms as well. This method can directly tackle whole-life degradation data, with no need to manually mark fault data in advance. Therefore, this method is more applicable for the online detection of early fault, and the idea of this paper can be widely expanded for different objects.

In the next work, an attention mechanism will be introduced into domain adversarial training to improve the effect of domain adaptation for time series data. Besides, this paper focuses on the anomaly detection problem for a bearing across different working conditions. How to achieve online transfer learning across different machinery and extract common features from multiple sources is an interesting problem. 

## Figures and Tables

**Table 1:** Description of the three working conditions in IEEE Prognostics and Health Management (PHM) Challenge 2012 dataset.

Operating Condition	Rotating Speed (rpm)	Radial Force (N)
First operating condition	1800	4000
Second operating condition	1650	4200
Third operating condition	1500	5000

**Table 2:** State assessment results of the IEEE PHM Challenge 2012 dataset.

Bearing	Normal State Period	Fault State Period
Bearing1_1	[1–1371]	[1372–2803]
Bearing1_2	[1–716]	[717–871]
Bearing1_3	[1–1165]	[1166–2375]
Bearing1_4	[1–931]	[932–1428]
Bearing1_5	[1–2235]	[2236–2463]
Bearing1_6	[1–1587]	[1588–2448]
Bearing1_7	[1–2067]	[2068–2259]

**Table 3:** Comparative results of the proposed method with nine state-of-the-art methods. Earlier detection location and lower number of false alarms indicate better. BEMD-AMMA, bandwidth empirical mode decomposition-adaptive multi-scale morphological analysis; LOF, local outlier factor; TCA, transfer component analysis.

Type of Methods	Methods	Bearing2_1		Bearing2_4	
		Detection Location	Number of False Alarms	Detection Location	Number of False Alarms
Signal analysis	1. BEMD-AMMA [35]	185	–	748	–
Anomaly detection without transfer learning	2. LOF [36]	610	40	746	137
	3. iFOREST [37]	307	35	745	152
	4. SRD [38]	870	12	745	13
Online anomaly detection without transfer learning	5. SDFM [1]	175	0	744	0
	6. S4VM-SODRMB [17]	763	10	744	11
Anomaly detection with transfer learning	7. TCA+SVDD	615	21	744	46
Online anomaly detection with transfer learning	8. RD-DTL [39]	175	0	744	0
	9. OD -DTL [26]	169	5	744	3
	10. **DTDA**	**162**	3	**744**	**0**

## Data Availability

The data presented in this study are openly available in PCoE Datasets at https://ti.arc.nasa.gov/tech/dash/groups/pcoe/prognostic-data-repository/, reference number [34].
